# Pre-operative embolization and surgical resection of extracranial superficial arteriovenous malformations

**DOI:** 10.1186/s42155-025-00606-2

**Published:** 2025-10-16

**Authors:** Shankar Rajeswaran, Abhinav Balu, Joe Baker, Joseph R. Ness, Darshan Variyam, Ali Shaibani, James Donaldson, Akira Yamada

**Affiliations:** 1 Division of Interventional Radiology, Ann & Robert H. Lurie Children’s Hospital, Northwestern University Feinberg School of Medicine, Chicago, IL USA; 2 Northwestern University Feinberg School of Medicine, Chicago, IL USA; 3 Division of Plastic Surgery, Ann & Robert H. Lurie Children’s Hospital, Northwestern University Feinberg School of Medicine, Chicago, IL USA

## Abstract

Extracranial arteriovenous malformations (AVMs) are high-flow vascular malformations that are challenging entities to treat and are primarily managed with embolization alone. Single-stage embolization and resection is a technique occasionally used for treating intracranial arteriovenous malformations. This manuscript describes combined embolization and surgical resection for localized superficial extracranial AVMs by presenting three cases from our experience. A retrospective review of the medical records of patients in this series including clinical notes, procedural imaging, and follow-up documentation was performed. A total of three AVMs near the antecubital fossa, deltoid, and forehead were embolized and resected. There was no evidence of recurrence on follow-up imaging, and no complications were observed, specifically, overlying skin injury. Embolization and resection of localized superficial extracranial AVMs with overlying skin changes offer an alternative treatment option to existing paradigms.

**Supplementary Information:**

The online version contains supplementary material available at 10.1186/s42155-025-00606-2.

## Introduction

Arteriovenous malformations (AVMs) are high-flow vascular malformations defined by abnormal connections between the arteries and veins without capillary involvement. These lesions can cause localized tissue necrosis, skin ulceration, or hemorrhage, with the potential to develop high-output cardiac failure [1]. There is currently no standardized treatment paradigm for extracranial AVMs, and options include embolization and, less frequently, surgical resection. Incomplete embolization has been associated with AVM proliferation and angiogenesis, possibly due to hypoxia or inflammatory-mediated angiogenesis, which often necessitates multiple procedures [2]. Surgical resection has also been associated with intraoperative hemorrhage and lesion proliferation, potentially via a similar mechanism [3, 4]. Embolization utilizing liquid embolics such as Onyx™ or sclerosants such as ethanol can cause skin ulceration and tissue necrosis [1], the risk of which increases in superficial AVMs with overlying skin involvement. In addition, colored liquid embolics such as Onyx™ will cause skin discoloration with superficial lesions; thus, alternative treatment paradigms would be beneficial in this patient population.

Intracranial AVM management with a multidisciplinary approach of pre-operative embolization and subsequent resection has been described as a more effective treatment modality than embolization or resection alone [5, 6]. This joint approach reduces intraoperative bleeding and helps border demarcation, increasing the likelihood of a complete and curative resection [5]. This effective treatment paradigm for localized AVMs is not commonly performed extracranially; however, the concept of pre-operative embolization and resection of venous malformations has been gaining traction in recent years [7]. Existing literature on this technique for extracranial AVMs primarily relates to the head and neck, with limited reports on peripheral AVM management. In this series, we present three separate patients with superficial extracranial AVMs, two peripheral and one scalp lesion, who underwent pre-operative embolization by interventional radiology prior to surgical resection with plastic surgery. Superficial AVMs are challenging lesions to treat, and this case series demonstrates that pre-operative embolization and resection is a viable and potentially curative treatment approach.

## Materials and methods

A comprehensive review of the medical records of three patients that underwent pre-operative embolization and resection was performed with Institutional Review Board (IRB) approval from our institution. Clinical presentation, pre-operative consultations, operative notes, pathology reports, and follow-up documentation were collected and analyzed.

## Results

### Case 1

A 13-year-old male presented with a painful enlarging lesion of the ventromedial aspect of the upper arm near the antecubital fossa with overlying skin changes. Magnetic resonance imaging (MRI) and an ultrasound were obtained, with imaging findings most suggestive of an arteriovenous malformation. The lesion was well localized, and, given overlying skin involvement, pre-operative embolization and surgical resection were performed.

**Fig. 1:**
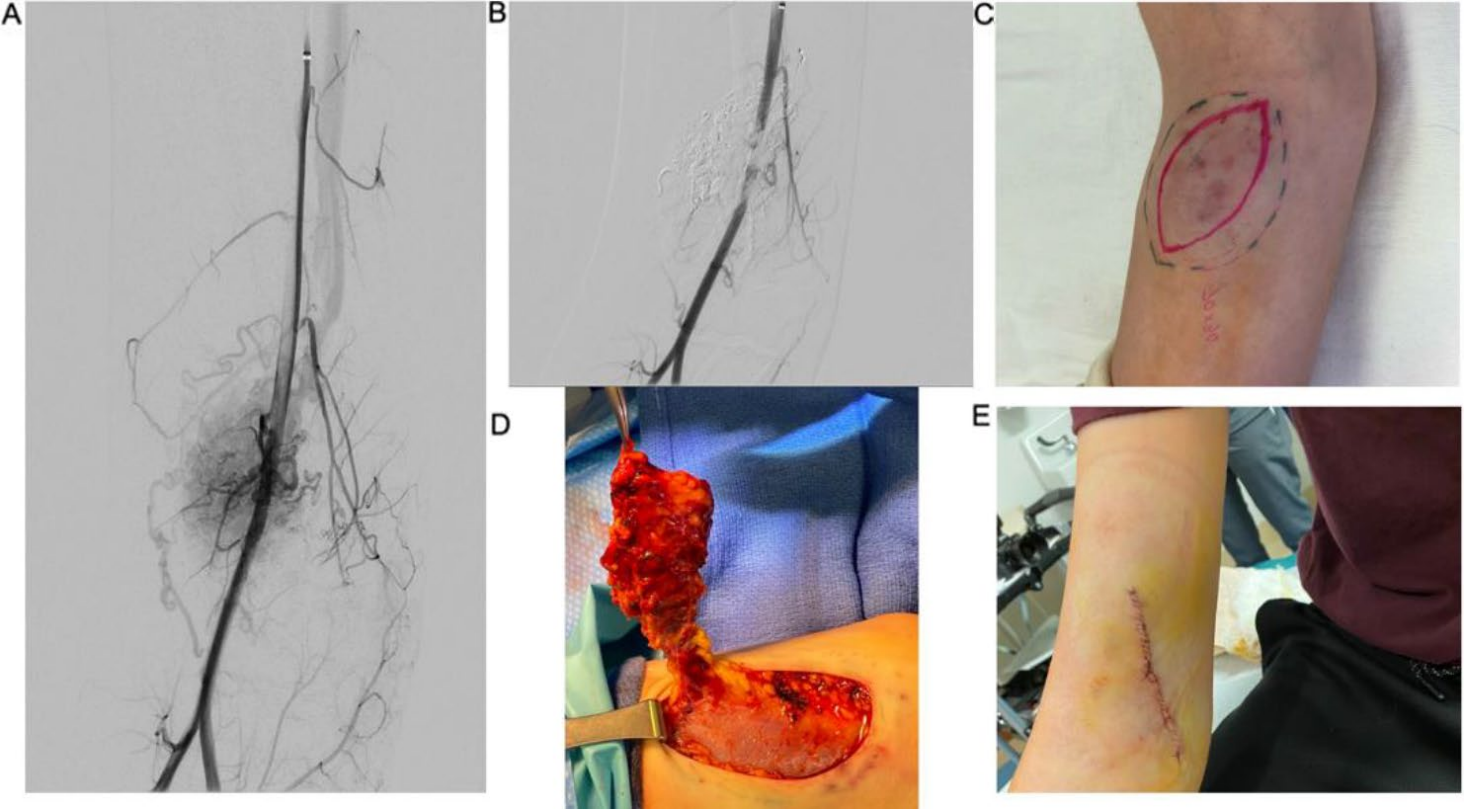
Right upper extremity AVM with overlying skin changes. **A** An angiogram from the brachial artery showing an arteriovenous malformation centered just above the antecubital fossa. **B** Angiogram post-embolization with N-BCA via endovascular and a percutaneous approach shows significant decrease in shunting. **C** Estimated margin of lesion and planned excision line. **D** Excision of AVM. **E** Primary closure of the excision site

Initial right brachial arteriography demonstrated an AVM (Yakes type 4) near the antecubital fossa (Fig. 1). The largest pedicles were embolized with 1.5 cc of a 1:2 N-butyl cyanoacrylate (NBCA) via microcatheters. Subsequently, the nidus was targeted percutaneously with a 21 g needle with a 1:3 NBCA to lipiodol ratio. Completion of right brachial arteriography revealed near-complete embolization with minimal residual vascularity.

The patient was then transferred under the same anesthetic to the operating room (OR) where a 70 mm × 30 mm elliptical skin incision was performed. The malformation was estimated to measure 10 cm × 5 cm. Following dissection, six vessels were ligated with 4–0 Prolene above the fascial plane, and the excised lesion and overlying skin were sent to pathology. Primary closure was achieved. There was an estimated 10-mL blood loss and no postoperative complications. Pathology results were consistent with an AVM, and the most recent follow-up ultrasound performed at 32 months showed no residual disease.

### Case 2

**Fig. 2:**
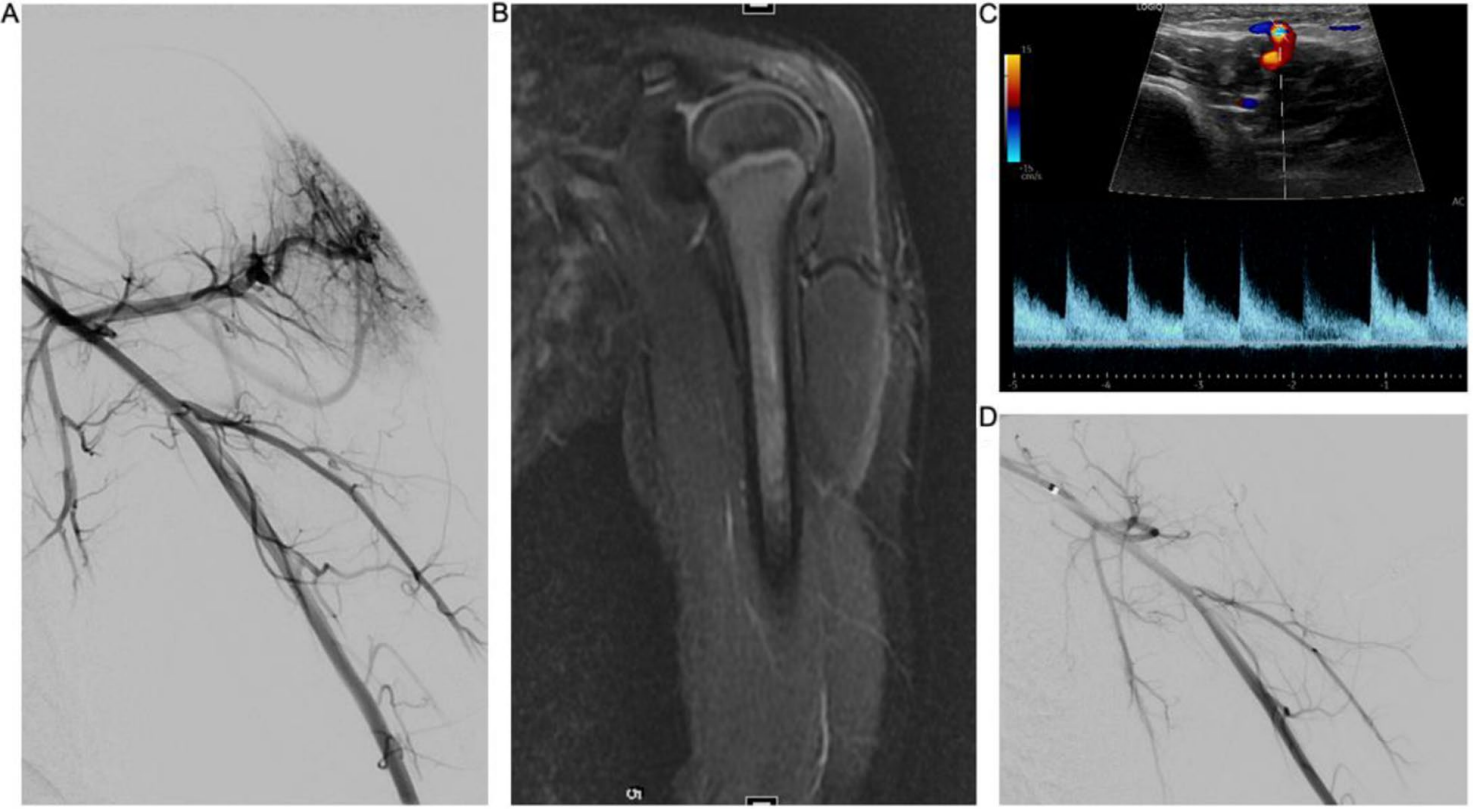
Shoulder AVM with overlying skin changes. **A** An angiogram from the axillary artery showing an arteriovenous malformation centered over the deltoid. **B** T2 coronal fat sat MRI showing a vessel traversing the deltoid with smaller branches in the subcutaneous tissue. **C** Ultrasound shows a large arterial pedicle traversing the deltoid and feeding branches in the subcutaneous tissue. D. Angiogram post-embolization with N-BCA from the axillary artery

**Fig. 3:**
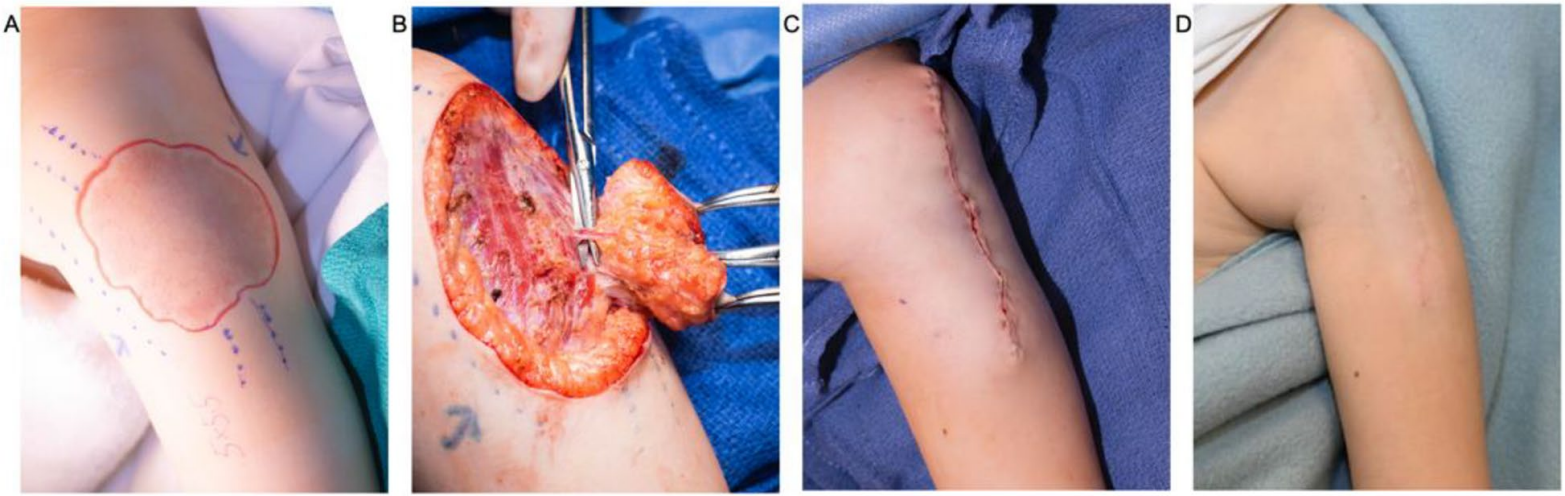
Intraoperative images from Fig. 2 showing the AVM resection and follow-up. **A** Estimated margin of lesion and planned excision line. **B** Excision of AVM. **C** Primary closure of the excision site. **D** Six-month clinic follow-up

A 4-year-old female presented with a vascular malformation on the left deltoid, which was originally diagnosed as a non-involuting hemangioma. Due to increasing growth, discomfort, and warmth of the lesion, she was then referred to interventional radiology by dermatology for further evaluation and possible treatment (Figs. 2 and 3). An ultrasound and MRI were performed, with imaging findings suggestive of an AVM. Embolization alone was deferred because of the concern for skin necrosis due to overlying skin involvement; therefore, a combined embolization with excision was pursued.

Under general anesthesia, an angiogram demonstrated an AVM (Yakes type 4) centered over the left deltoid. A 1:1 NBCA to lipiodol mixture was administered into the main feeding artery via a microcatheter, just proximal to the malformation. The post-embolization angiogram demonstrated minimal blood flow into the AVM.

The anesthetized patient was then transported to the OR for resection. Excision began with a 1–2-mm margin around the erythematous area of the lesion. Capillary-like vessels were ligated, and a monopolar needle point was used to excise the lesion above the muscle, and a primary closure was performed. There was 3 mL of intraoperative blood loss post-embolization. There were no postoperative complications. The most recent follow-up ultrasound performed at 30 months showed no residual disease. A departmental consensus conference reviewed the specimen and determined the pathology to be consistent with an arteriovenous malformation.

### Case 3

A 7-year-old boy with a vascular lesion with overlying skin involvement centered over the forehead presented with pain and cosmesis concerns to plastic surgery. An MRI confirmed the diagnosis of an AVM. Under general anesthesia, angiography revealed that the left superficial temporal artery was hypertrophied and was the predominant inflow vessel of the AVM (Yakes type 2) with a small contribution from the left ophthalmic artery. Venous drainage revealed was primarily via the ophthalmic vein and the external jugular system. Ultrasound guidance was utilized to puncture the nidus with a 21-gauge needle, and a 1:3 NBCA-lipiodol mixture was injected. Repeat angiography showed no residual flow to the AVM.

**Fig. 4:**
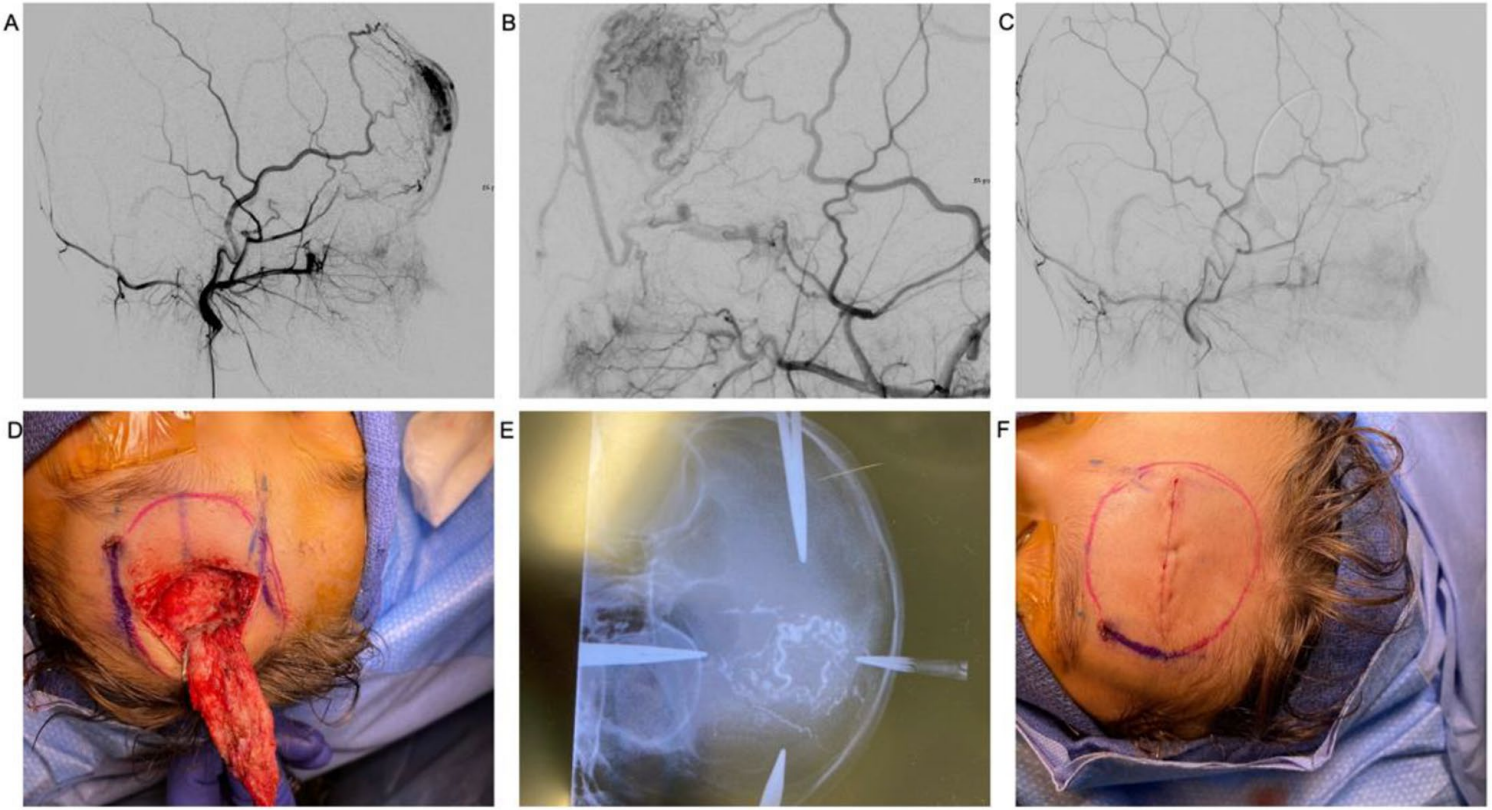
Left forehead AVM with overlying skin changes. **A** An angiogram via the external carotid artery showing an arteriovenous malformation centered over the left forehead. **B** Magnified view showing multiple arterial pedicles feeding the AVM. **C** Angiogram post-embolization with N-BCA via a percutaneous approach. **D** Excision of AVM. **E** Intraoperative radiograph performed for nidus localization. **F** Primary closure of the excision site

The resection was performed the next day under a second anesthetic due to a scheduling conflict that precluded same-day excision. The following day, the patient was placed under general anesthetic, and a transverse 4.5-cm incision was designed across the forehead. Soft tissue and overlying frontalis muscle were dissected to reveal the AVM (Fig. 4). The draining veins were identified and ligated with 4–0 nylon prior to complete excision. 5–0 Vicryl was used to close the frontalis muscle prior to primary wound closure with 5–0 PDS. The patient tolerated the procedure well with minimal blood loss and no intra- or postoperative complications. Pathology results were consistent with an AVM, and a follow-up angiogram performed at 6 months showed no residual AVM.

## Discussion

This case series highlights three separate cases in which pre-operative embolization and subsequent surgical resection were successfully utilized to treat extracranial AVMs with overlying skin involvement. Follow-up imaging of all three lesions show no evidence of recurrence, and given the overlying diseased skin was resected, no complications related to skin ulceration secondary to embolization were encountered. The approach of pre-operative embolization and resection to localized AVMs is not novel and well described in the intracranial literature with multiple studies showing that this approach allows for significantly improved clinical outcomes [8, 9]. In addition to improving the clinical outcomes, combined embolization and resection had a significantly shorter resection time compared to surgical resection alone, reducing the likelihood for intraoperative complications and blood loss [10].

Combined embolization and resection of AVMs has been demonstrated to be a safe and effective procedure for intracranial lesions; however, this approach is not widely used in the management of extracranial AVMs. Surgical resection does increase the overall invasiveness when compared to embolization alone; however, the ability to eradicate the nidus, which is centered within the excised tissue, is the hallmark of curing an AVM. A unique aspect of extracranial AVMs is the ability to percutaneously access the nidus, which is often performed by identifying the nidus based on fluoroscopy and correlating it with a sonographic target, which is then subsequently accessed using sonographic guidance.

Choosing the appropriate patient that will benefit from extracranial single-stage embolization and resection is multifactorial. The lesion needs to be superficial such that the underlying musculature is not involved, and the lesion should be small and localized to allow primary closure without a skin graft. During embolization, because the nidus will be resected, the primary goal is to de-vascularize the lesion. If the nidus cannot be adequately targeted, such as with more infiltrative AVMs, the main feeding artery can be embolized (Case 2). It should be noted that such an approach would not be a viable treatment option if endovascular treatment alone was chosen, as the nidus would remain untreated. The author’s preference is to not perform any form of venous compression during embolization due to the risk of altering flow dynamics and potentially refluxing embolic into normal arterial branches.

Attempting to perform the embolization and resection under a single anesthetic has the primary benefit that skin injury is of limited concern, as it will be immediately resected, thus allowing an operator to be more aggressive, and not having to manage patient discomfort or ulceration if it were staged. In addition, post-embolization inflammation is often most significant at 48 h, which can obscure surgical planes during a resection. Lastly, the ability to limit general anesthetics is often appealing to patients, especially in the pediatric population. The authors acknowledge that not all centers will have the ability to consolidate anesthetics, and as demonstrated with the third case, a staged resection is also a viable option.

Our early experience demonstrates that pre-operative embolization and resection of extracranial AVMs represents an alternative approach to embolization alone, with the possibility of being curative and avoiding repeat interventions.

## Supplementary Information

Supplementary Material 1.

## Data Availability

Data sharing is not applicable to this article as no datasets were generated or analyzed during the current study.
