# Supplementary figures and images for: Pre-operative embolization and surgical resection of extracranial superficial arteriovenous malformations
Source: CVIR Endovasc. 2025 Oct 16;8:90. doi: 10.1186/s42155-025-00606-2 (PMC12528573; doi:10.1186/s42155-025-00606-2)

## Slide 1
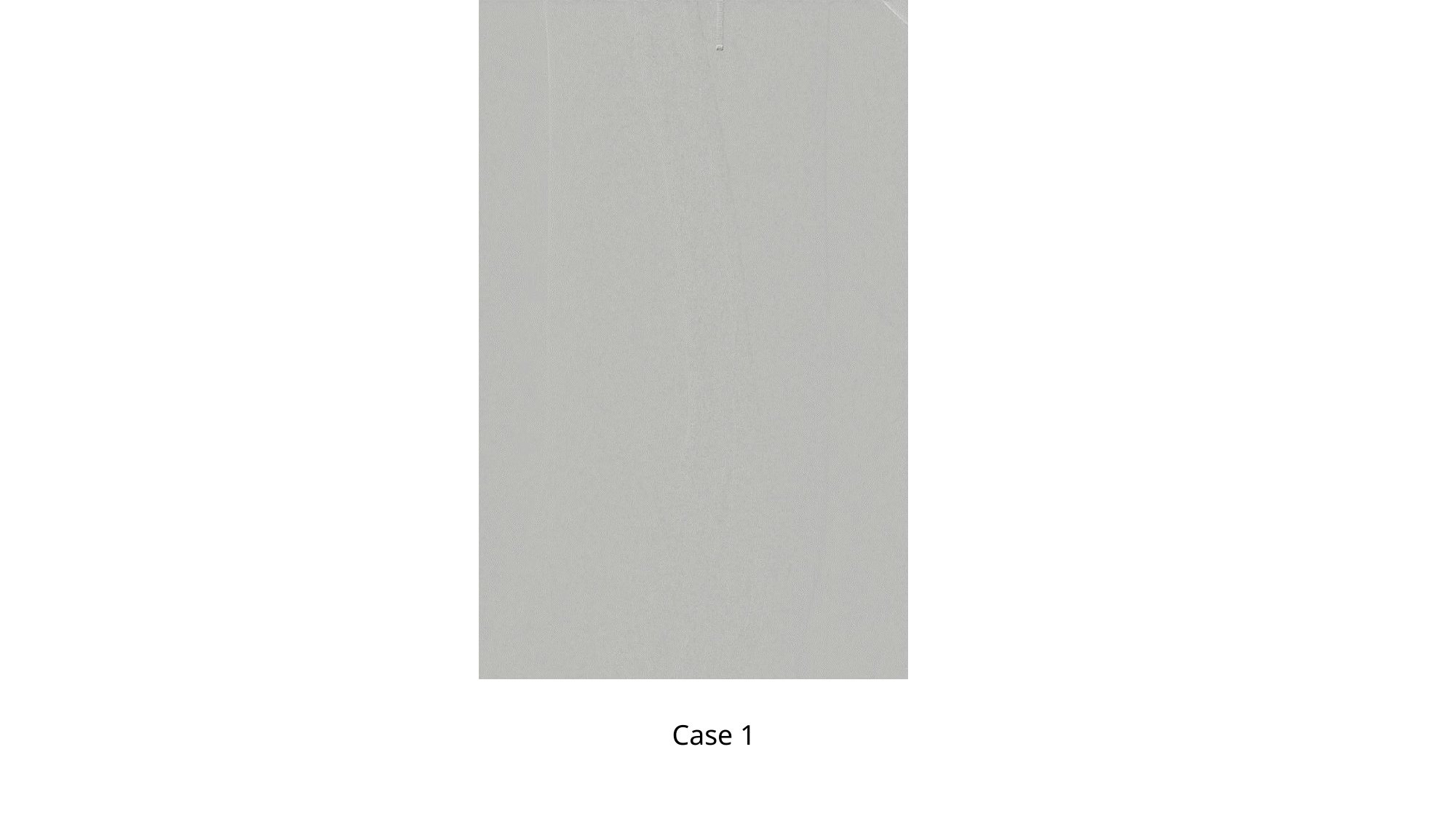

Case 1

## Slide 2
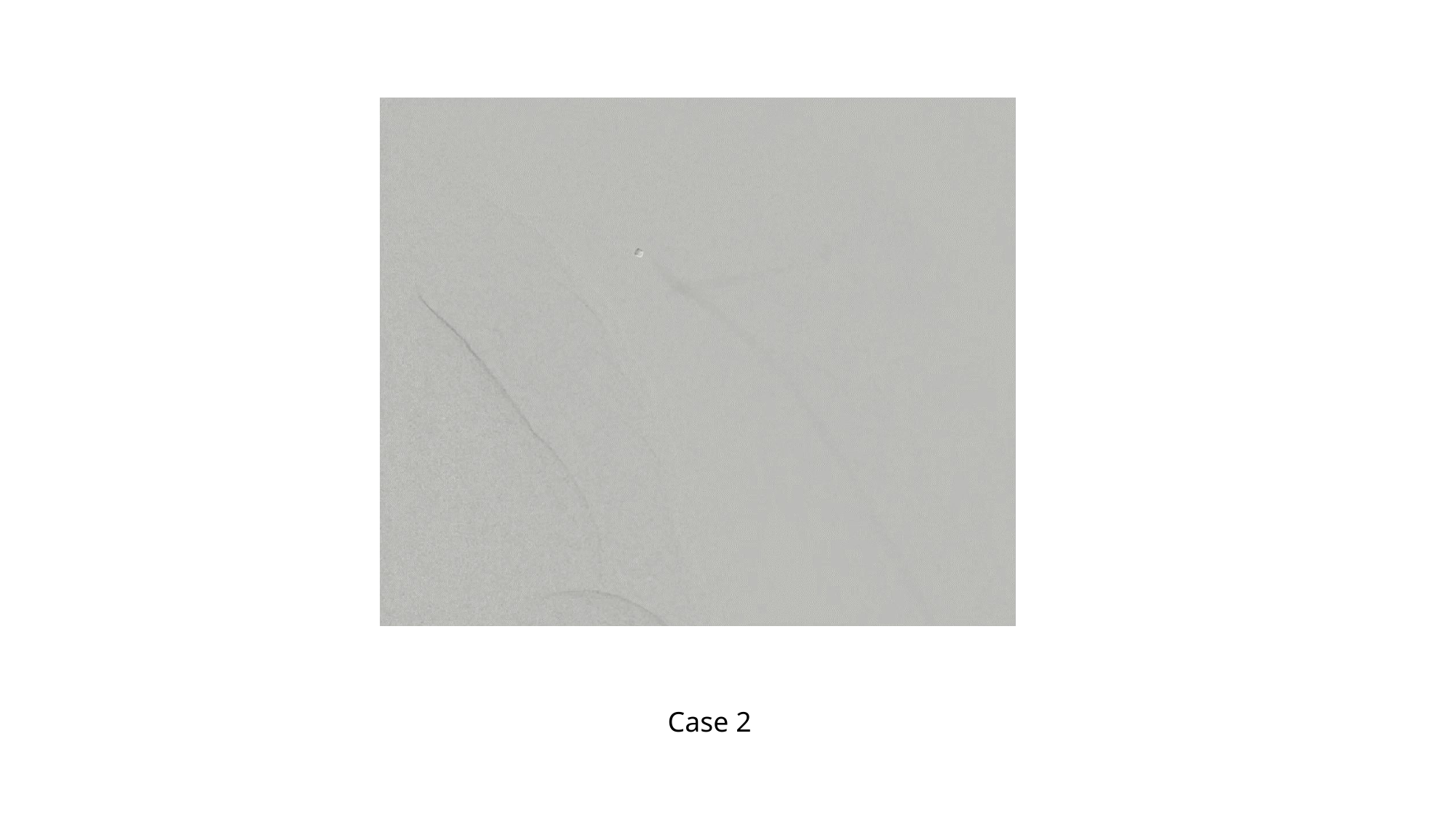

Case 2

## Slide 3
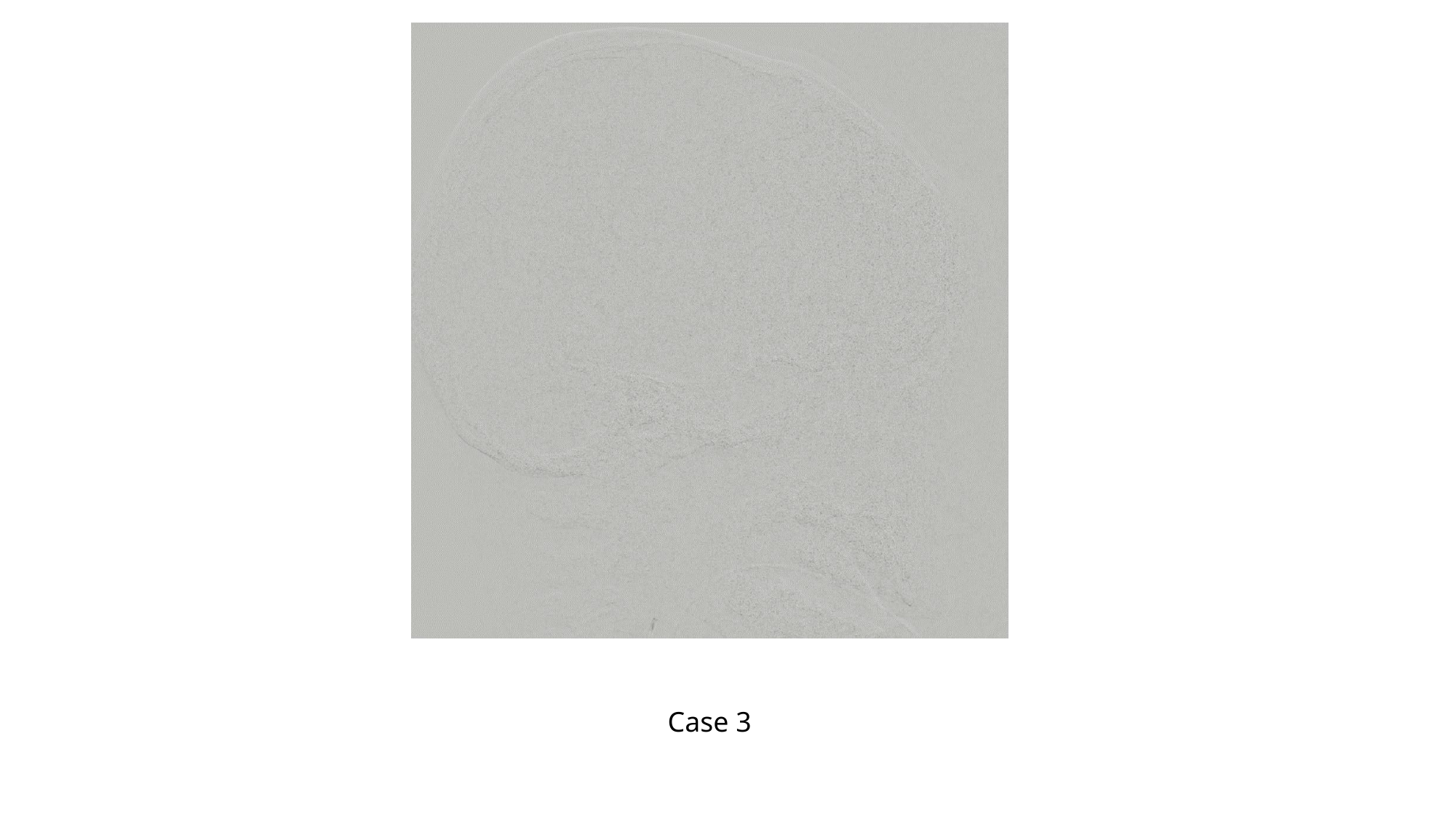

Case 3

Supplement: Supplementary file 1 — Supplementary Material 1. [file 42155_2025_606_MOESM1_ESM.pptx]
